# Study Habits of Medical Students: An Analysis of which Study Habits Most Contribute to Success in the Preclinical Years

**DOI:** 10.15694/mep.2018.0000061.1

**Published:** 2018-03-12

**Authors:** Jenny Liles, Jasna Vuk, Sara Tariq

**Affiliations:** 1 University of Tennessee Health Sciences Center; 2 University of Arkansas for Medical Sciences

**Keywords:** study habits, USMLE preparation, medical student education

## Abstract

This article was migrated. The article was marked as recommended.

Academic performance during the first two years of medical school is an important predictor of success on the United States Medical Licensing Exam Step 1. Research is lacking into what study methods successful students use, with success being defined as achieving a grade point average of above 90% in all or in most of the courses in the preclinical years.This study sought to identify specific study habits that successful students use and to demonstrate an association between preclinical grades and Step 1 scores. In this study, an anonymous survey was sent to first, second, and third year medical students that included various questions about their study habits, as well as their course grades (A, B, C, or fail) and, if applicable, their Step 1 score. Results demonstrated statistically significant differences existed between Step 1 scores and grades in the second year of medical school, with A students earning higher scores. A students tended to attend class, limit use of online lectures, study for 6-8 hours a day, and review lectures the same day they were given significantly more than B and C students did. This study demonstrates that certain study habits are employed consistently by successful students. These study habits should be shared with medical students early in the preclinical years to help students reach maximum potential both in class and on Step 1, which in turn will allow students to match into their choice of residency.

## Introduction

Medical school is an exceptionally challenging and rigorous academic endeavor. Though almost all students who are accepted into medical school were at the top of their class in college, medical school represents unique challenges to students due to the sheer volume and breadth of information such that students who previously did well academically may be forced to find new ways to study effectively. Determining effective ways to study in medical school is of paramount importance, since preparation for the United States Medical Licensing Exam (USMLE) Step 1 begins on day one of medical school. Therefore, an understanding of the types of study habits that are most effective in medical school is crucial, as knowing early which study habits are associated with success can help students to reach their full potential during the preclinical years and perform well on the Step 1 examination.Performing well on Step 1 is critical as it is a major determining factor in the residency selection process ( Dort et al., 2015; Go, Klaassen, & Chamberlain, 2012).

While there has been research into the types of learning approaches ( Arnold & Feighny, 1995), the social habits of successful students (Ogenler & Selvi, 2014), and study habits during dedicated USMLE Step 1 study time ( Kumar et al., 2015), research is lacking about the specific study methods usedduring the first two years of medical school. Abdulghani et al. (2014) used focus group discussion to qualitatively analyze habits of successful studentsand found certain themes that emerged. In particular, we were interested in what types of resources and study habits students use during the first two years of medical school, and in turn how effective these resources and habits are, measured by how well the students who use them perform in class. Grades were reported as A, B, C, or fail, with A corresponding to a grade of 90% or above, B to an 80% or above, C to a 70% or above and fail being below a 70%. This information is critical, as the grades that are made during the first two years (particularly the second year) have been shown to be predictive of Step 1 scores as well as of success in the clinical years ( Hu et al., 2016; West,Kurz, Smith, & Graham, 2014).

The first goal of this study was to investigate the differences between students’ grades during the first two years of medical school and their performance on Step 1. As noted above, previous studies have found significant differences ( Coumarbatch, Robinson, Thomas & Bridge, 2010; Hojat, Gonnella, Erdmann, & Veloski,1996; Hu et al., 2016; Swanson, Ripkey, & Cas,1996; West et al., 2014; Zhang, Rauchwarger, Toth, & O’Connell, 2004), so this project sought to corroborate these claims. The second goal of this study was to identify study habits that successful students used, with successful students being defined as those who made mostly or all A’s. Several specific study habits were of interest. The first was class attendance versus use of recorded lectures. Online recorded lectures are becoming more and more popular, and there are studies that suggest students who attend class receive limited if any benefit in terms of academic performance ( Eisen et al., 2015; Hammen & Kelland, 1994; Vidler 1980). However, other studies suggest that attending class results in significantly better outcomes ( Gunn,1993; Nyamapfene, 2010; Park & Kerr, 1990; Romer, 1993; Subramaniam, Hande, & Komattil et al.,2013; Von Blerkon, 1992). Since not in all of these studies participants were medical students, we wanted to see specifically if medical students who made coming to class a priority performed better on exams. Related to this idea, we also wanted to know if re-watching the lectures posted online was a habit associated with success.

Another habit of interest was the note-taking strategies of medical students. Many students choose to take notes on laptops rather than by hand, often because notes taken on a computer offer increased speed and legibility compared to those taken by hand ( Kim,Turner, & Pérez-Quiñones, 2009). However, a recent study suggested that handwriting notes results in a better understanding of the material ( Mueller & Oppenheimer, 2014), so we wanted to know if students who primarily took notes by hand performed better in class. Otherstudy habits examined included when successful students reviewed lectures (i.e., same day versus later), how many hours a day students spent studying, as well as how many and what type of outside resources successful students used. By investigating these study habits, we hoped to identify trends that separated the successful students (students who made mostly or all A’s) from less successful ones (students who made mostly C’s). With a better understanding of what study habits are most effective, faculty mentors and educators will ideally be better able to assist students who are struggling and offer students concrete suggestions for altering their study habits to ones that have proven to be effective.

## Methods

### Participants and Instrument

A survey was designed using SurveyMonkey software that required the student to first give their grades for the past year (i.e. 1 A, 3 B’s, 1 C, etc.) and then went on to questions about how they study-do they attend class, do they take notes, what resources do they use, etc. Third year medical students were asked to answer the survey based on their grades from their second year, and they were also asked to report their first attempt Step 1 score. Participants were recruited via an email with the link to the survey and brief explanation of the survey’s purpose and goals. The invitation to the survey was sent to current first (M1), second (M2) and third (M3) year medical students in our institution Approximately 500 students received the survey and 206 responded (41% response rate). Any surveys that were incompletely answered were removed from the data set prior to analysis. The study was submitted to the University of Arkansas for Medical Sciences IRB (protocol number 205452) for approval and was found to be exempt.

### Data Analysis

Once data was collected, results from SurveyMonkey were imported to Excel and transferred to IBM SPSS-22 program. Descriptive statistical analysis, chi-square tests for independence, and one way Analysis of Variance (ANOVA) were conducted. When assumptions of chi-square statistics were not met and expectedfrequency of any cell was less than 5, exact chi-square and Monte Carlo chi square statistics were conducted. Monte Carlo chi square statistics was computed with 10 000 sampled tables, starting seed 2 000 000 and 99% Confidence Interval (CI) for a true p value ( Gravetter & Wallnau, 2007; Mehta & Patel, 2010). Cramer’s V was used to measure effect sizes or strength of relationships among categorical variables ( Kotrlik & Williams, 2003). Participants’ self-reported performance in the last year (A’s, B’s, C’s, and below C’s) were used to place students in 1 of 4 groups. Students who made all A’s were placed in the all A group. Students who made a majority of A’s were placed in the mostly A group. Students who made a majority of B’s were placed in the mostly B group. Students who made a majority of C’s were placed in the mostly C group. Successful students were defined as those who made either all A’s or mostly A’s. Percents (frequencies) of students’ study habits, note taking, use of outside resources, and lectureattendance were reported for the entire group and separately divided into categories based on students’ grade average during their most recent year of medical school. With regard to the reported Step 1 scores, differences between students’ grades during the second year of medical school and score on first attempt USMLE Step 1 were analyzed using One-way ANOVA. For the purpose of this analysis, students were divided into an all A group, mostly A group, mostly B group, and 1 or more C group.

## Results

### Second Year Grades and Step 1 Scores

Results demonstrated that students who made all or mostly A’s during their M2 year earned higher Step 1 scores than students who made mostly B’s or 1 or more C. One-way ANOVA revealed a statistically significant difference among the four groups, with F(3)=18.09, *p*< 0.01, and effect size of 0.32. Bonferoni post hoc test was conducted and showed a statistically significant difference among all groups except between the group who made mostly B’s and the group who made 1 or more C (see Table 1).

**Table 1.** Means and Standard Deviations of USMLE Step 1 Scores of Students in Four Groups

			95% Confidence Interval for Mean	
	Mean Score	Std Dev	Lower Bound	Upper Bound
All A’s	246.68	13.233	240.31	253.06
Mostly A’s	233.58	11.524	228.92	238.23
Mostly B’s	220.86	16.529	211.31	230.4
1 or more C’s	211	14.908	200.12	221.88

### Class-Related Study Habits

With regard to study habits, results showed a number of statistically significant associations of study habits and students’ success ( Table 2). Physically attending class was associated with making all or mostly A’s ( *p* = 0.001; see Table 2). Twenty-six (52%) students who made all A’s reported going to class 5 days a week, whereas 55 students (63%) who made mostly B’s and 6 students (75%) who made mostly C’s reported going to class 2 days a week or less. Twenty-four students (42%) who made mostly A’s went to class 3 days a week or more (see Figure 1).

**Table 2.** Chi squared values, degrees of freedom, p values, effect size, and sample size (n) for results associated with statistical significance.

	Class Attendance	Use of Online Lectures	Re-watching Lectures	Study Hours	Same-day Review of Lectures	Use of Najeeb (online lecture series)
χ ^2^	26.94	24.30	27.69	28.03	13.91	9.946
df	9	9	9	9	6	3
p	0.001	0.003	0.001	0.001	0.034	0.019
Effect Size	0.211	0.2	0.214	0.216	0.184	0.220
n	202	202	202	201	206	206

**Figure:**
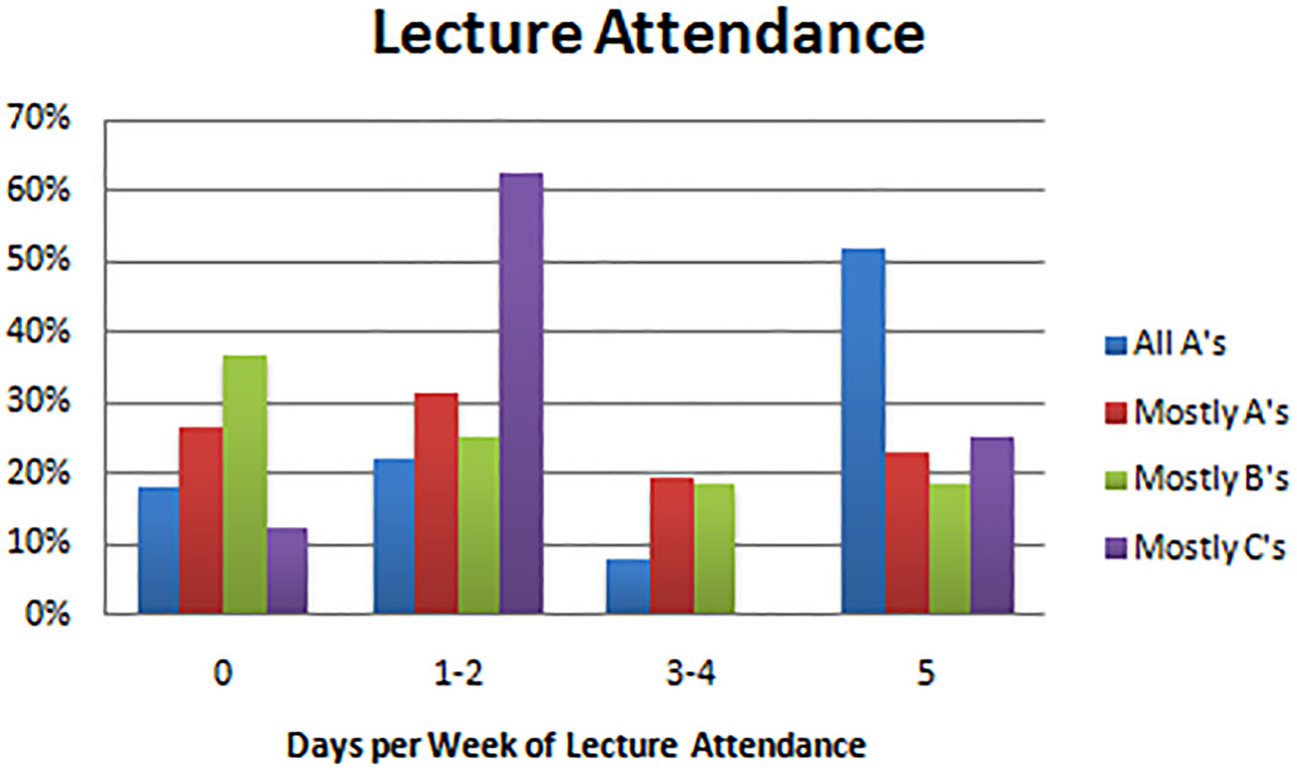


Results demonstrated that students who made all A’s tended not to watch lectures recorded online, as 27 (54%) all A students reported no use of online lectures. B students, on the other hand, were more likely to watch online lectures, as only 23 (26%) reported no use of online lectures. Students who made mostly C’s were much more likely to watch the lectures online, with 5 (63%) students using them more than 3 times a week. Students who made mostly A’s were evenly split regarding use of online lectures, as 30 (54%) of them reported using online lectures at least 3 times a week. Associations were statistically significant with *p* = 0.003 (see Table 2, Figure 2).

**Figure:**
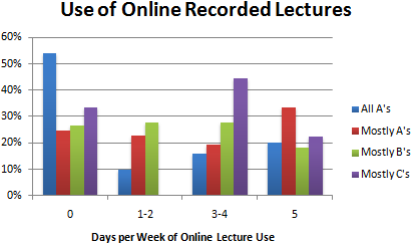


Re-watching lectures was not a habit employed often by successful students, as 22 (44%) all A students never re-watched lectures and the other 21 (42%) all A students only reported re-watching lectures one to two times per week. Similarly, 28 (49%) of students who made mostly A’s never re-watched lectures, and 19 (33%) re-watched lectures only once or twice a week. This was in contrast to the C students, where 5 (63%) reported re-watching lectures five days per week. Associations were statistically significant with estimated *p* =0.001 (see Table 2, Figure 3).

**Figure:**
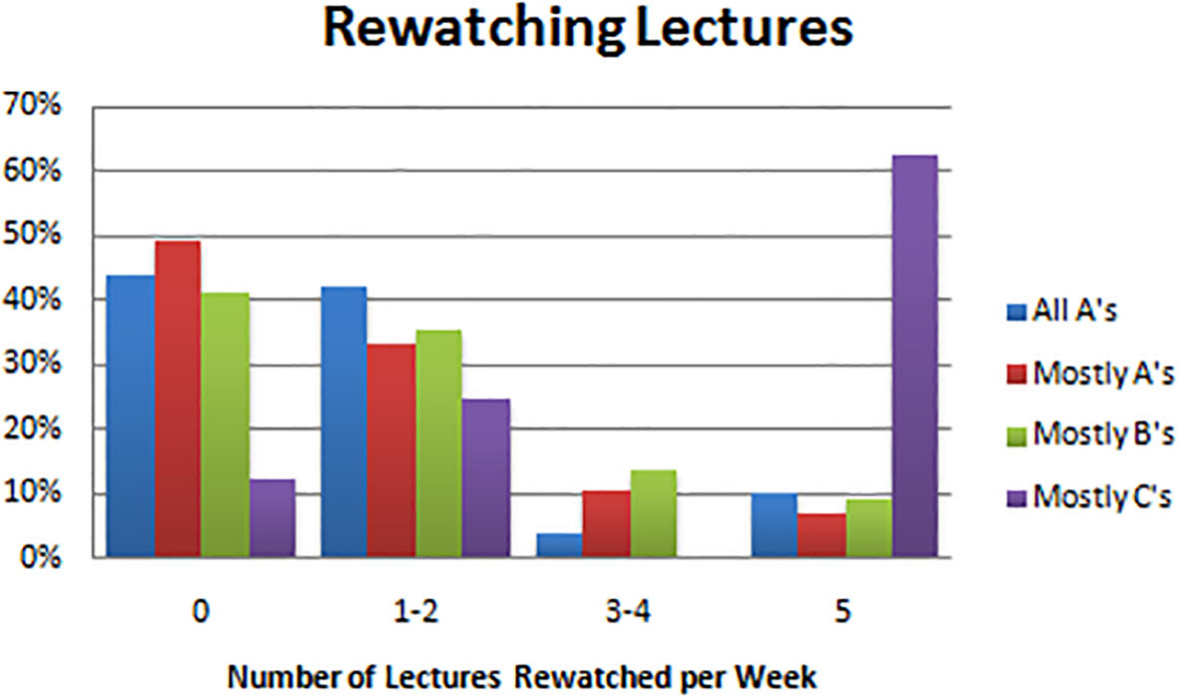


### Out of Class Study Habits

Results indicated that 25 (50%) all A students studied 6-8 hours a day. This is in contrast to the B and C students, who studied on average 3-5 hours per day as 45 (52%) majority B students and 5(63%) majority C students fell into the same category. Students who made mostly A’s fell in the middle, with 24 (42%) studying more than 6 hours a day and 28 (49%) studying 3-5 hours a day. Studying more than 8 hours a day was not associated strongly with success, as only 9 (18%) all A students and only 5 (9%) mostly A students were in that category. Associations were statistically significant with estimated *p* =0.001 (see Table 2, Figure 4).

**Figure:**
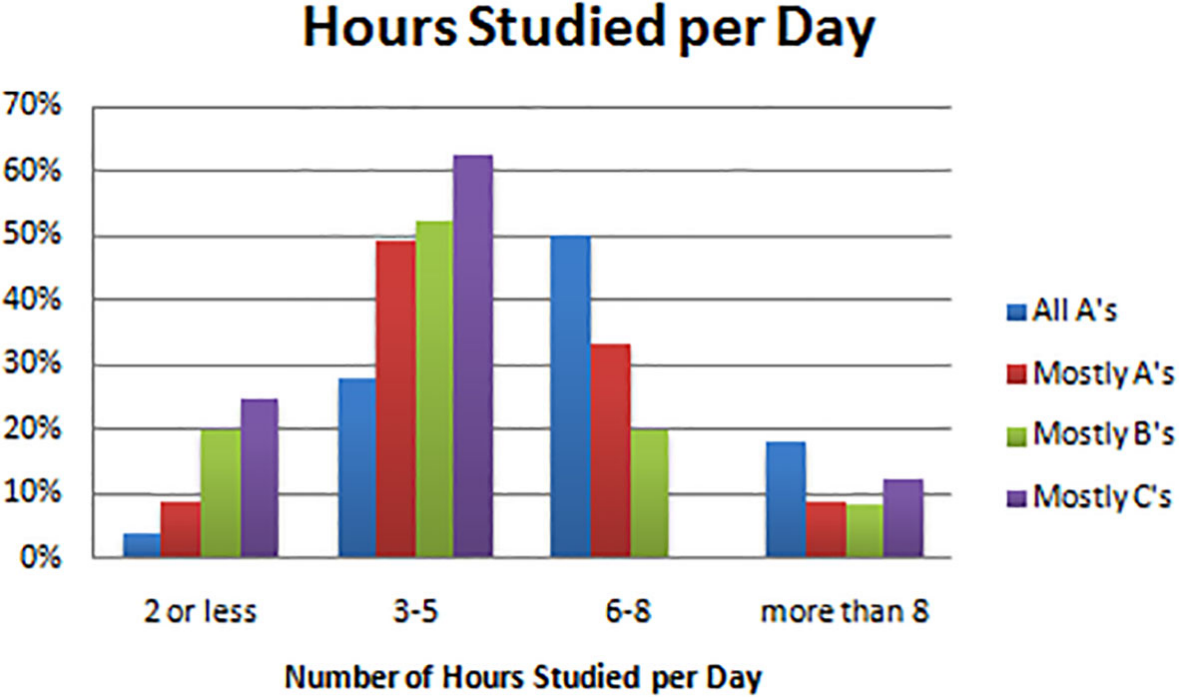


Same-day review of lecture material was shown to be a successful habit, as 39 (77%) students who made all A’s and 39 (66%) students who made mostly A’s reviewed the lecture the same day, whereas only 2 (25%) students who made mostly C’s and 49 (56%) mostly B students reported reviewing lectures the same day they were given.These associations were statistically significant with estimated *p*=0.034 (see Table 2, Figure 5).

**Figure:**
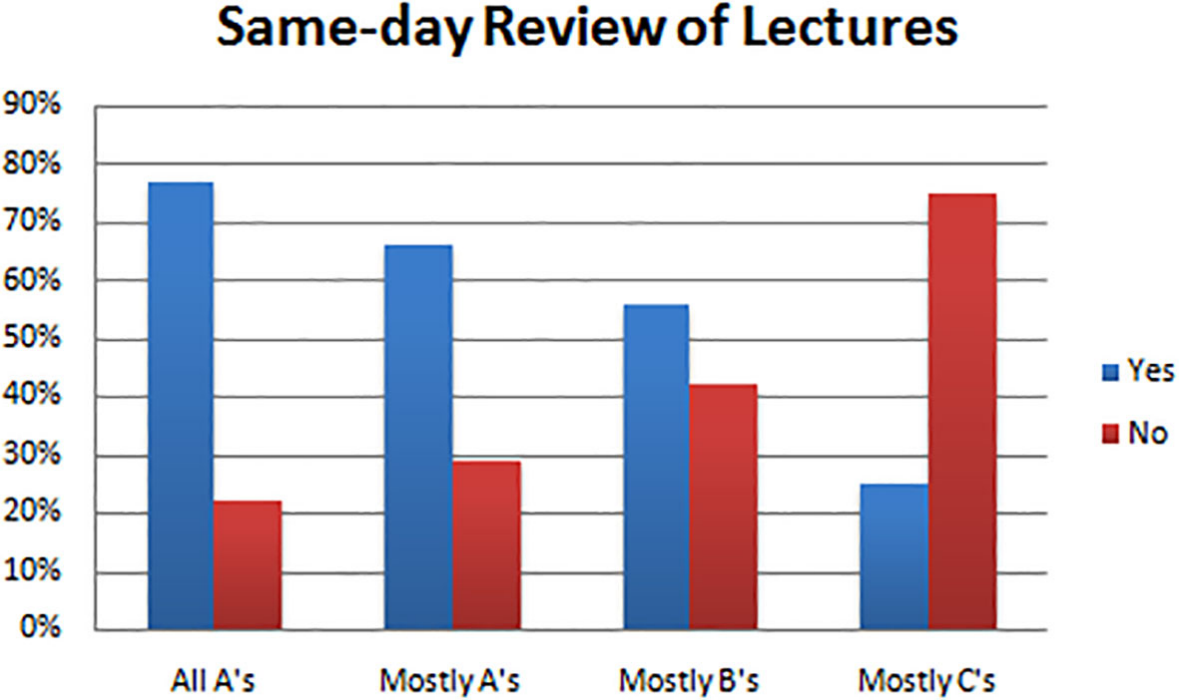


Also statistically significantly different between A students and other students was whether or not they were able to cover all lectures during their home study time prior to the test. Forty-two (84%) students with all A’s reviewed all lectures while only 3 (38%) C students were able to review all the lectures. These results had an estimated *p*=0.029 (CI 99% 0.024-0.033) and moderate effect size (Cramer’s V=0.236).

Finally, most students reported handwriting their notes without much difference in success. Thirty-four (68%) A students, 29 (52%) mostly A students, 50 (57%) mostly B students, and 6 (75%) mostly C students reported doing so. These results were not associated with statistical significance.

### Use of Outside Resources

Most of the data regarding the use of outside resources did not reach statistical significance but did demonstrate interesting trends. The most commonly reported resources used were First Aid ( Le & Bhushan, 2015) and Pathoma (Sattar, 2011), with greater than 75% of students from all groups reporting consistent use of these resources. Using an increasing number of resources was not associated with success, as thirty-five (69%) students who made all A’s used only 1-2 resources, while 4 (50%) C students used 3-4 resources. Additionally, students who made A’s tended to not use UWorldQuestion Bank for study during the preclinical years, as only 11 (22%) all A students reported use of UWorld. It was also not necessary to do assigned reading for classes to be successful, as 34 (71%) all A students reported “rarely” or “never” doing the assigned reading. Interestingly, the resource that was associated with unsuccessful studying was online video lecture series such as *Dr. Najeeb Lectures.*Four (50%) mostly C students reported consistent use of online lectures by Dr. Najeeb, compared to 4 (8%) A students, 11 (19%) mostly A students, and 20 (23%) mostly B students. This association was statistically significant with an estimated *p* of 0.019 (99% CI 0.016-0.023) and moderate effect size (Cramer V=0.200) (see Table 2).

## Discussion

Overall, results indicated that there were certain study habits that were associated with success while other study habits were not. One especially important result of the study was the difference between Step 1 scores and grades in the preclinical years. Our results echo similar results found by other investigators who also have noted this link ( Coumarbatch et al., 2010; Hojat et al.,1996; Swanson et al.,1996; West et al., 2014; Hu et al.,2016;Zhang et al., 2004). Students should be told early on in their 1 ^st^ year that Step 1 is a critical component of their residency application, and that the material they learn in the first two years (as reflected in their pre-clinical grades) will have an impact on how well they do on Step 1. Our data supports the idea that time spent studying for classes during the first two years prepares students for success on Step 1.

### Class-Related Study Habits

Attending class was strongly associated with success. These results echo previous studies which have also reported increasing academic performance when students attend class ( Gunn, 1993; Nyamapfene, 2010; Park & Kerr, 1990; Romer, 1993; Von Blerkon, 1992). Subramaniam et al. (2013) found significantly increased grades when medical students were required to attend class. Similarly, the qualitative study by Abdulghani et al. (2014) also found that successful students reported attending class, so our results provide quantitative evidence to corroborate this claim. In a world where technology is becoming increasingly more prominent and medical schools are facing pressure to offer all lectures online, results of our study suggest that there is still value in attending a lecture. It should be noted, though, that students who are willing to wake up and go to class may also be the students who are most motivated to study, which could partly explain why they are more successful than the students who chose to watch lectures from home. Interestingly, in Romer’s study (1998) factors such as student motivation were controlled, but even when controlling for these exogenous factors, students who came to class still performed better than those who did not, which suggests that attending lecture is of value in and of itself.

Alternatively, one habit employed by many students who made C’s was frequently re-watching lectures online as part of their studying; students who made more A’s almost never reported re-watching lectures. This data suggests that re-watching a lecture is not sufficient to learn the material. A better approach might be to actively engage in the material by taking notes, studying in groups, etc. rather than passively watching. It should be noted that students who were successful did report occasional use of re-watching lectures, but only 1 to 2 times per week. This is in sharp contrast to the C students, who reported re-watching lectures every day. The overall conclusion is that while it may be helpful to re-watch parts or all of lecture that were poorly understood, this should not be a primary way of studying, and it should not replace physically attending lecture.

### Out of Class Study Habits

Results of our study also indicated that students who were studying about 6-8 hours a day outside of class were the most successful overall. Of note, only a small proportion of students who made all A’s reported studying more than 8 hours a day, which indicates that students should not feel obligated to forgo all other aspects of their lives in order to study. Previous studies have also noted a relationship between amount of time studying and success; for example, longer amounts of time spent studying correlated with higher GPA’s of college students when students reported being able to concentrate during their entire study period ( Nonis & Hudson, 2010). Our results support this idea, as it is likely that students who study longer than 8 hours a day are unable to fully concentrate for that long and thus do not derive added benefit from the extra time spent studying, whereas students who study for around 6 hours a day are able to concentrate the entire time. The mostly A students were split between studying 3-5 hours a day and studying 6-8 hours a day. Studying only 3-5 hours was the most popular category reported by the B and C students. One explanation may be that the B and C students tended to study closer to three hours, whereas the mostly A students tended to study closer to five hours.

A third study habit associated strongly with success was reviewing lectures the same day. Students who made A’s almost always reviewed the lecture the same day it was given, while C students almost never did so. In fact, the higher a student’s grades were, the more likely they were to report reviewing the lecture the day it was given. These results echo findings in a recent study that replicated Ebbinghaus’s forgetting curve, which demonstrates the value in reviewing material soon after it is given in order to maximize future ability to recall the information ( Murre & Dros, 2015). Abdulghani et al. (2014) also reported that students who performed well in class frequently reviewed lectures the same day they were given. Following along closely with this data is the fact that students who made A’s were able to review all lectures before the test, while a strikingly less number of C students were able to do so. These results suggest that successful students are successful because they go to class and then spend the following 6 or so hours reviewing the lectures from that day. If they do this every day, they are never behind, and thus they review all lectures before a test. On the other hand, C students (and majority B students as well), tend more often not to go to class and do not study enough during the day to review all the lectures prior to the test, resulting in lower scores.

The fact that handwriting notes was not more strongly associated with success was an interesting result of this study, as handwriting notes is a more active study method than simply reading or listening, and previous studies ( Fink, 2010; Mueller & Oppenheimer, 2014; Piolat, Olive, & Kellog, 2005; Stacy & Cain, 2015) have indicated that handwriting notes can be beneficial for long term learning and performance on conceptual questions. So, we anticipated that A students might be more likely to handwrite their notes, but instead, a majority of students in all groups chose to handwrite their notes. One potential explanation for this result is the small number of C students in the study; had there been more C students, perhaps we would have seen a trend of lower grades corresponding to less handwriting of notes. It is also possible that students can be equally successful by typing their notes if they type in a way that forces them to actively engage with the material, such as by making concept maps, mind maps,and charts ( Alton, 2016; D’Antoni, 2010). Further studies are needed to determine if there are differences in how A students versus B and C students take notes by hand, and if taking notes on a laptop can be equally effective.

### Use of Outside Resources

A final trend regarding successful students that emerged was that these students tended to limit their use of outside resources to 1-2 well known and well established resources such as Pathoma and First Aid. Interestingly, most A students did not report using the UWORLD question bank to study during the school year. UWORLD’s question bank is a very popular resource for Step 1 studying, but the fact that students who make A’s are not using it all throughout the year to study-and yet still perform well on Step 1-indicates once again that studying for class by reviewing the lectures given by faculty is effective preparation for Step 1, and the use of UWORLD throughout the year is not strictly necessary. A large percentage ofstudents who made mostly C’s reported the use of outside video lectures while a much smaller proportion of A students used such resources. While online video lectures resources may be valuable adjuncts to study, as evidenced by the fact that a proportion of successful students used them, these results indicate that placing too much emphasis on online videos is not an effective study strategy, as it likely takes time away from reviewing the material presented in class.

## Limitations

While this study did demonstrate clear trends regarding study habits, it is important to remember that what works for one student may not work for another, so students should also be encouraged to find study habits that work well for them. The habits presented here are meant to be a guide that may be of assistance to students whose current study habits are not working well, as we present some concrete examples of ways to do well in class. It should also be noted that our Step 1 scores and pre-clinical year grades were self-reported, which introduces the possibility of error secondary to inaccurate reporting. Also, students who elected to respond to the survey may have been the students who performed better in class and on Step 1, which could have skewed our data. The low number of C students in the study suggests this possibility as well. Additionally, results are only from survey responses collected at one school, so they may not be representative of medical students across the country. It should also be noted that while significant differences emerged between Step 1 scores and class grades, these trends are not absolute. While the preclinical years are invaluable in laying the foundation for Step 1, the time students spend on exclusive Step 1 studying also likely impacts students’ score, and this study did not address study habits used during dedicated Step 1 study time.

## Conculsions

Results of this survey of 206 medical students indicate both that performance in the pre-clinical years can predict success on Step 1, and that there are certain study habits that almost all successful students use. Students who go to class and spend 6-8 hours studying daily with use of only a select few outside resources tend to perform better in class. In contrast, students who do not go to class, spend time re-watching lectures online and using multiple outside resources (including online video series) do not perform nearly as well in class. Successful students also make it a habit to study the material the same day it is given, resulting in them not falling behind. Lower performing students spend only 3-5 hours a day studying are more likely to not review lectures the day they are given, which makes it easier for them to fall behind and not cover all material before the test. The results of this study have implications both for medical students and for their educators and mentors. Ideally these results can be used to help guide medical students towards effective study habits early on in their medical education, which will allow them to reach their full potential on Step 1and beyond.

## Take Home Messages

• Grades during the first two years of medical school are important predictors of success on USMLE Step 1. Study habits of students who are successful in their preclinical courses is therefore of interest to medical educators.
• Students who make all or mostly A’s attend class, limit use of online lectures and outside resources, study for 6-8 hours a day, and review lectures the same day they are given.
• Students who make more B’s and C’s are more likely to not attend class, make frequent use of online lectures, study for 3-5 hours a day, and not review lectures the same day they are given.
• These study habits should be shared with medical students and medical educators alike so that students can maximize their full potential in the preclinical years, which in turn will help them perform well on Step 1 and match into a residency of their choice.

## Notes On Contributors

Jenny Liles, M.D. is currently completing her preliminary year of training in Internal Medicine at the University of Tennessee for Health Sciences Center in Memphis, TN. Next July she will began her residency in Dermatology at Medical College of Georgia in Augusta, GA. She has a passion for medical education and hopes to continue to make teaching a part of her career.

Jasna Vuk, M.D, Ph. D. is an Associate Professor in the Division of Academic Affairs, Student Success Center at the University of Arkansas for Medical Sciences.

Sara Tariq, M.D., is the Assistant Dean for Undergraduate Clinical Education and Associate Professor in the Department of Medicine at the University of Arkansas for Medical Sciences.
