# Supplementary material for: Study Habits of Medical Students: An Analysis of which Study Habits Most Contribute to Success in the Preclinical Years
Source: MedEdPublish (2016). 2018 Mar 12;7:61. [Version 1] doi: 10.15694/mep.2018.0000061.1 (PMC10712003; doi:10.15694/mep.2018.0000061.1)
Supplement: Peer review history

# Reviewer report: Reviewer response for version 1

**DOI:** 10.21956/mep.19527.r28902

**Published:** 2018-04-09

**Authors:** Alyss Robinson

**Affiliations:** 1 University of Leeds

This review has been migrated. The reviewer awarded 4 stars out of 5 This was a simple yet highly insightful retrospective cohort study assessing patterns of study behaviour in relation to grades as an outcome. The findings are interesting, and help to discourage the philosophy that the pre-clinical years “don’t matter”. The primary outcome was to assess whether pre-clinical grades were congruent with final exam performance, which they were. Secondary outcomes aimed to analyse the efficacy of study habits, namely to identify superior methods so that students “may be forced to find new ways to study effectively”, in order to “reach their full potential”. The main findings highlighted that students scoring the highest grades in exams seemed to attend lectures, review lectures and spend 6-8 hours studying outside of scheduled teaching. It would be valuable if the authors described the examination schedule in greater depth; how many examinations do students take? How often? How are they weighted? This is important because a student may consistently score C grades in smaller frequent examinations but one A grade in a major end-of-year examination, for example. This would also help with generalisability. Also are the distributions of grades of study participants comparable to the medical school, or could there be a potential survey bias?The authors speculate that students achieving A grades are attending lectures, and then spending 6-8 hours reviewing the lectures from that day at home. It would be useful if they had elucidated how students were spending their private study time from the survey. This would further the study as it would suggest that thoroughly covering the lecture material is key for exam success, which is one theory postulated in the discussion.One minor grammatical comment; there should be no apostrophe when describing grades, it should be As, Bs and Cs not A’s, B’s and C’s. The definition of a successful student was a student achieving all or mostly A grades. While not within the realms of this study design, more reflection and discussion was required to emphasise that successful students are not limited purely by exam performance. More so in later years, students may be furthering their academic profile; publishing papers, attending conferences and teaching students which would occupy time otherwise spent studying. However, these activities would make the student more successful in other domains. More reflection was also needed on potential reporting bias. The numbers of students within each group would be useful to enable the reader to assess the sample sizes. The median/mean hours studied or the absolute number of lectures attended/watched/caught-up per week would be more useful than the categories provided to statistically compare groups, however this is understandably difficult to elucidate in a questionnaire and would be better assessed in prospective work. Overall this was a very interesting read which evokes interesting thought and discussion. Thank you.

Reviewer Expertise:

NA

No decision status is available

# Reviewer report: Reviewer response for version 1

**DOI:** 10.21956/mep.19527.r28905

**Published:** 2018-03-16

**Authors:** Juan Cendan

**Affiliations:** 1 University of Central Florida College of Medicine

This review has been migrated. The reviewer awarded 5 stars out of 5 This is an important contribution to the current medical education literature. Just this week, an article was published ahead of print in Academic Medicine touting a universal (and proximate) medical education curriculum platform. It is critical that medical educators come to grips with the avalanche of technological advances that are competing for students' attention and that we engage with these and understand how to curate these resources while complementing the experience for our learners. The paper is well-written. The overall numbers are good; but warrant corroboration at multiple sites. The information is very useful.

Reviewer Expertise:

NA

No decision status is available

# Reviewer report: Reviewer response for version 1

**DOI:** 10.21956/mep.19527.r28904

**Published:** 2018-03-16

**Authors:** PATRICIA CURY

**Affiliations:** 1 FACERES

This review has been migrated. The reviewer awarded 4 stars out of 5 This is a very interesting work! I wonder how the sutdents study 6-8 hours daily. Is it out of the university ? In my contry medical course is from 8 AM to 6 PM. Was the study only with Med students from University of Arkansas? How long they spend their time in class? Is it PBL?

Reviewer Expertise:

NA

No decision status is available

# Reviewer report: Reviewer response for version 1

**DOI:** 10.21956/mep.19527.r28901

**Published:** 2018-03-13

**Authors:** Julie Hunt

**Affiliations:** 1 Lincoln Memorial University

This review has been migrated. The reviewer awarded 4 stars out of 5 This retrospective study analyzes self-reported student data about their study habits and correlates those with their grades. The graphs in the paper beautifully describe what the statistics showed: increasing grades with lecture attendance and daily review of lecture material, and decreasing grades with use of online lecture viewing to replace in-person attendance and re-watching of lectures. Like some of the other studies on this topic, it offers further support for what many of us academics have been telling our students. I agree with the previous reviewer who pointed out a number of confounders exist. Given that it was a retrospective study, it shows association but not causation. For example, the students who missed daily lectures and relied on online lectures may be those who also have family, job, or health issues that take away from their study time.

Reviewer Expertise:

NA

No decision status is available

# Reviewer report: Reviewer response for version 1

**DOI:** 10.21956/mep.19527.r28903

**Published:** 2018-03-12

**Authors:** John Cookson

**Affiliations:** 1 University of Worcester

This review has been migrated. The reviewer awarded 4 stars out of 5 This is an interesting study but as with similar reports, there may be a confounder at work. Thus B and C students may have other issues which are reflected both in their study habits and their grades. Adoption by them of the study habits of A students (even assuming they are able to do so) may not lead to improvement.

Reviewer Expertise:

NA

No decision status is available
